# Myricitrin inhibits fibroblast-like synoviocyte-mediated rheumatoid synovial inflammation and joint destruction by targeting AIM2

**DOI:** 10.3389/fphar.2022.905376

**Published:** 2022-08-31

**Authors:** Chuyu Shen, Meilin Xu, Siqi Xu, Shuoyang Zhang, Wei Lin, Hao Li, Shan Zeng, Qian Qiu, Liuqin Liang, Youjun Xiao, Hanshi Xu

**Affiliations:** ^1^ Department of Rheumatology and Immunology, The First Affiliated Hospital, Sun Yat-sen University, Guangzhou, Guangdong, China; ^2^ Department of Rheumatology, The First Affiliated Hospital of Jinan University, Guangzhou, China

**Keywords:** AIM2, rheumatoid arthritis, aggression, inflammation, myricitrin, fibroblast-like synoviocytes

## Abstract

**Objective:** To explore the effect and underlying mechanism of Myricitrin (Myr) in regulating fibroblast-like synoviocyte (FLS)-mediated synovitis and joint destruction in RA.

**Methods:** FLSs were isolated from synovial tissues from patients with RA. Gene expression was measured using quantitative RT-qPCR. Protein expression was detected by immunohistochemistry or Western blot. Cell apoptosis was performed by an Annexin-PI staining assay. EdU incorporation was used to assess the proliferation of RA FLS. Transwell assay was used to characterize the cell migration and invasion ability of RA FLS. The potential target of Myr was identified by RNA sequencing analysis. The *in vivo* effect of Myr was assessed in a collagen-induced arthritis (CIA) model.

**Results:** Myr treatment inhibited the lamellipodia formation, migration, and invasion, but not the apoptosis and proliferation, of RA FLSs. Myr also reduced the expression of CCL2, IL-6, IL-8, MMP-1, MMP-3, and MMP-13 induced by TNF-α. The RNA-seq results indicated that AIM2 may be a target gene of Myr in RA FLSs. Furthermore, compared to healthy controls, AIM2 expression showed higher levels in synovial tissues and FLSs from RA patients. AIM2 knockdown also inhibited RA FLS migration, invasion, cytokine, and MMP expression. In addition, either Myr treatment or AIM2 knockdown reduced the phosphorylation of AKT induced by TNF-α stimulation. Importantly, Myr administration relieved arthritis symptoms and inhibited AIM2 expression in the synovium of CIA mice.

**Conclusion:** Our results indicate that Myr exerts an anti-inflammatory and anti-invasion effect in RA FLSs and provide evidence of the therapeutic potential of Myr for RA.

## Introduction

Rheumatoid arthritis (RA) is a systemic autoimmune disease characterized by chronic synovial inflammation and aggressive destruction of joint, the etiology of which is not yet fully understood. The basic pathological features of RA are pannus formation, synovitis, and bone destruction. Fibroblast-like synoviocytes (FLSs), which are in a continuously activated state and have the characteristics of “tumor-like cells”, including secreting proinflammatory cytokines and exhibiting aggressive behavior to cartilage and bone (Ai et al., 2018; Yoshitomi, 2019), are a key factor in RA synovial inflammation and joint destruction. Increasing evidence suggests that selective treatment targeting RA FLS may hold promise for ameliorating rheumatoid joint damage (Nygaard and Firestein, 2020).

At present, the main treatment drugs for RA include glucocorticoids, nonsteroidal anti-inflammatory drugs (NSAIDs), disease-modifying anti-rheumatic drugs (DMARDs) including conventional synthetic DMARDs, biological DMARDs, and targeted synthetic DMARDs. Although DMARDs have significantly improved the prognosis in patients with RA, their clinical application is restricted due to insufficient effectiveness, high cost and potentially high toxicity (Guo et al., 2018). Therefore, there is still a need to find alternative therapeutic drugs for RA.

Natural compounds from herbal medicines have been discovered and successfully used to treat RA patients. These drugs have the advantages of wide availability of sources, relatively low prices, multiple targets, multistep effects, etc., and have become an important source and component of RA treatment drugs (de Seabra Rodrigues Dias et al., 2021).

Myricitrin (Myr) is a well-known natural bioactive small molecular flavonoid that is widespread in fruits, vegetables, and medical herbs (Hobbs et al., 2015). Myr has several biological effects including anti-atherosclerotic (Gao et al., 2019), anti-viral (Jo et al., 2020), and hepatoprotective efficacy (Omidi et al., 2020). Previous studies have shown that Myr has strong antioxidant activities by attenuation of oxidative stress and regulation of the inflammatory response through the AMPK/SREBP-1c pathway in liver cells (Gao et al., 2018). Furthermore, Myr significantly inhibits VEGF-induced angiogenesis of human umbilical vein endothelial cells (Hu et al., 2020). In addition, Myr has a potential effect on osteoarthritis, preventing the collagen-II degradation of chondrocytes by inhibiting the activation of NF-κB and MAPK in response to IL-1β(Yan et al., 2020). However, whether Myr has a therapeutic effect on RA remains unclear.

Therefore, we evaluated whether Myr has an effect on the apoptosis, proliferation, inflammation, migration, and invasion of RA FLS. Furthermore, we delineated the potential mechanisms of Myr on RA FLS function and its properties in collagen-induced arthritis (CIA) mice.

## Methods

### Materials

Myr was purchased from Aladdin (Shanghai, China). Myr was dissolved in DMSO (Sigma, St Louis, MO, United States). Fetal bovine serum (FBS), DMEM, trypsin-EDTA (0.25%), and PBS were purchased from Gibco (NY, United States). TNF-α (human recombinant) was obtained from Peprotech (Rocky Hill, NJ, United States). Anti-β-tubulin antibody was obtained from Sigma (St Louis, MO, United States). Anti-AIM2, anti-AKT, and anti-phospho-AKT (Ser473) antibodies were obtained from Cell Signaling Technology (Beverly, MA, United States). Anti-AIM2 antibody for IHC was purchased from Abcam (Cambridge, MA, United States). Anti-IL-6, anti-IL-8, anti-CCL2, anti-MMP-3 and anti-MMP-13 antibodies were obtained from Affinity Biosciences (Jiangsu, China), anti-MMP-1 antibody were obtained from Proteintech (Wuhan, China). Anti-CD11b, anti-FcγRII, anti-FcγRIII and isotype control antibodies were purchased from Biolegend (CA, United States). Red fluorescent beads (particle size: 2 μm) were obtained from Zhongkeleiming (Beijing, China).

Collagen-Type II and complete Freund’s adjuvant were purchased from Chondrex (Woodinville, WA, United States). All materials used in this study were endotoxin-free.

### Human synovial specimens and FLSs preparation

Synovial specimens were collected from active RA patients (2 males and 8 females, aged 56.67 ± 4.63 years) undergoing arthroscopic knee joint synovectomy. RA patients fulfilled the 2010 revised criteria of the American College of Rheumatology/European League Against Rheumatism classification criteria. We obtained healthy control (HC) synovial specimens from 10 subjects (7 males and 3 females, aged 37.5 ± 13.33 years, 8 of them received NSAIDs treatment before surgery) who underwent traumatic above-knee amputation and without acute or chronic arthritis history.

Synovial tissues were cut into small pieces with scissors, placed on culture dishes for 2 h to allow cell to attach, and then cultured in DMEM medium supplemented with 10% FBS. FLSs of passages 4 to 6 were used in our experiments, which were a homogeneous population of cells (<1% CD11b positive, <1% FcγRII and FcγRIII receptor-positive, and <1% phagocytic, see Supplementary Figure S5). The medical Ethical Committee of the First Affiliated Hospital at Sun Yat-sen University approved the present study protocol (No. [2017]049), which was conducted in accordance with the recommendations of the Declaration of Helsinki. All study subjects agreed to participate in the study and provided written informed consent.

### RA FLS viability assay

Cell Counting Kit-8 (CCK8) assay kit (Dojindo, Kumamoto, Japan) was used to assess RA FLS viability. Briefly, RA FLSs were incubated with Myr at different concentrations (0–800 μM) for 24 h. Cells were washed with DMEM. Subsequently, a culture medium containing 10% Cell Counting Kit-8 (CCK-8) reagent was added to each well and incubated for another 4 h. Afterward, the absorbance of the plate was measured at 450 nm by a microplate reader.

### FLS migration and invasion assay

FLS migration was performed using a transwell assay with a filter (6.5 mm in diameter, 8.0 μm pore size) (Corning, Corning, NY, United States). Briefly, FLSs were suspended in serum-free DMEM in the upper compartments of the chambers at a concentration of 6×10^4^ cells/ml. Medium containing 10% FBS as a chemoattractant was added to the lower compartments of the chambers. After incubation for 8 h, the cells on the top surface of the membrane were scraped using a cotton swab. RA FLSs that migrated to the lower side of the filter were fixed in methanol for 15 min and stained with 0.1% crystal violet for 15 min. The number of stained FLSs is the average number of cells from 5 random fields. To measure cell invasion, we performed a similar experiment in chambers coated with BD Matrigel basement membrane matrix (BD Biosciences, Oxford, United Kingdom).

### FLS proliferation assays

RA FLS proliferation was measured by using a Cell-Light EdU DNA Cell Proliferation Kit (Ribobio, Guangzhou, China) following the manufacturer’s protocol. The cells were pretreated with different concentrations of Myr (0–400 μM) for 24 h. EdU was added to measure the cell proliferation and incubated for another 12 h. DAPI was used to stain cell nuclei. EdU-positive cells were quantified by microscopy.

### FLS apoptosis assays

An Annexin V-APC/PI Apoptosis Detection kit (BioGems, Westlake Village, CA, United States) was used to assess RA FLS apoptosis following the manufacturer’s protocol. Briefly, 1 × 10^5^ FLSs were suspended in 0.1 ml 1× binding buffer, stained with 5 μL PI and 5 μL annexin V and incubated for another 15 min in darkness at room temperature. The samples were then analyzed within 1 h by flow cytometry.

### Immunohistochemical staining

Synovial tissue sections (5 μm) were prepared, deparaffinized and rehydrated, then soaked in boiling retrieval solution (EDTA pH 9.0) for 15 min to recover the antigen. Sections were incubated with 3% H_2_O_2_ for 10 min to prevent endogenous peroxidase activity and then incubated with 5% BSA in PBS for 1 h to block nonspecific binding. The sections were stained with primary antibodies overnight at 4°C. For staining with secondary antibodies, enzyme-conjugated sheep anti-mouse/rabbit IgG polymer labeled with horseradish peroxidase (HRP) was added and continually incubated at room temperature for 20 min. The samples were revealed using diaminobenzidine.

### F-actin staining

RA FLS were cultured on 14 mm coverslips. Cells were fixed with 4% paraformaldehyde/PBS for 15 min, permeabilized with 0.1% Triton X-100/PBS for 5 min, and then blocked in blocking buffer for 1 h at room temperature. Cells were incubated with rhodamine-phalloidin (Sigma, United States) at room temperature for 30 min to detect F-actin. 4’,6-diamidino-2-phenylindole (DAPI, Shenggong, Shanghai, China) was used to label nuclei. Coverslips were mounted on glass slides by using Fluoroshield histology mounting medium (Sigma, United States) and examined by fluorescence microscope.

### Quantitative real-time PCR

Total RNA was isolated using TRIzol reagent (Sigma, United States). Real-time quantitative PCR (RT-qPCR) was performed to measure the levels of mRNAs by using SYBR Green Premix Pro Taq HS qPCR Kit (Accurate Biology, China). The primers used for real-time PCR are listed in Supplementary Table S1. RT-qPCR was then carried out on a Bio-Rad CFX96 system or a Roche LC480 system. The relative expression of each gene was calculated with normalization to β-actin by the 2^−ΔCT^ method (for Supplementary Figure S1A) or the 2^−ΔΔCT^ method (for other RT-qPCR results).

### Western blot analysis

Cells were dissolved in RIPA lysis buffer (Beyotime, Shanghai, China) supplemented with proteinase inhibitor cocktail and phosphatase inhibitor cocktail (Sigma, United States) for 15 min. Protein concentrations were determined by the bicinchoninic acid protein assay (Beyotime, China). Equal amounts of protein were solubilized in SDS-PAGE Sample Loading Buffer (Beyotime, China), boiled for 5 min, and then separated by SDS-PAGE. After transfer, the membranes were blocked with TBST buffer containing 5% nonfat milk, followed by incubation with primary antibodies overnight at 4 °C. After incubation, the membranes were incubated with the following anti-rabbit IgG or anti-mouse IgG (Cell Signaling Technology, United States) at room temperature for 1 h. The bands were visualized by ECL western blotting substrate (Millipore, MA, United States).

### siRNAs transfection

Small interfering RNA (siRNA) against AIM2 and negative control siRNAs were synthesized by RiboBio (Guangzhou, China). The siRNA sequences are shown in Supplementary Table S2. Cells were cultured at 70–80% confluence and transfected with siRNAs using Lipofectamine 3,000 reagent (Thermo Fisher Scientific, United States) following the manufacturer’s protocol. Experiments were performed at 48 h of siRNA transfection.

### RNA sequencing and data analysis

For RNA sequencing, total RNA was extracted by the TRIzol method and quantified using a NanoDrop ND-1000 instrument. cDNA library construction for RNA transcriptome sequencing was performed by Guangzhou Epibiotek Co., Ltd. (Guangzhou, China). Novel genes and transcripts were also predicted. Kyoto Encyclopedia of Genes and Genomes (KEGG) pathway, Gene Ontology (GO) enrichment analysis, and volcano plots were performed for the differentially expressed genes (DEGs) using R software and DAVID v6.8 (Huang da et al., 2009b; a) for statistical computing and graphics.

### CIA model and treatment

The CIA mouse model was established as previously described (Wang C. et al., 2020). Briefly, 7–8 weeks old male DBA/1J mice (Vital River Laboratory Animal Technology, Beijing, China) were intradermally injected with 100 μg of bovine type II collagen and Freund’s complete adjuvant in a 1:1 ratio (vol/vol) emulsion (100 μL) at the base of the tail. DBA/1J mice were intraperitoneally injected with 200 μg bovine type II collagen on Day 21 after the first immunization. In the therapeutic treatment study, the mice were randomly divided into groups. Myr group (100 mg × kg^−1^, every day, *n* = 5) or DMSO group (vehicle, *n* = 5) was intragastrically administered for 14 days. Arthritis progression was monitored every other day according to previously described scoring system ranging from 0 to 4 (0, normal; 1, swelling or redness of paw or a single digit; 2, 2 joints involved; 3, 3 joints involved; 4, severe arthritis of the entire paw and digits) (Thornton et al., 2017). The arthritic score was independently calculated in a blinded manner and defined as the combining scores of all four paws. All mice were raised under specific pathogen-free (SPF) conditions. All experiments were performed in accordance with institutional guidelines and approved by the Institutional Animal Care and Use Committee of the First Affiliated Hospital, Sun Yat-sen University (SYSU-IACUC-2020-000153).

### Hematoxylin and eosin and safranin O staining

The hind limbs of all mice were removed and fixed overnight in 4% paraformaldehyde, 0.5 M EDTA decalcified and paraffin embedded. H&E and Safranin O-Fast green staining were performed by Servicebio (Wuhan, China) in accordance with the standard protocol (GP1031, GP1051). Histological assessment of arthritis was evaluated on a 0 to 3 scale as follows: 0 (normal), 1 (mild), 2 (moderate) and 3 (severe) (Li et al., 2021).

### Measurement of serum biochemistry

To assess the toxic effect of Myr on CIA mice, serum glucose, creatinine and enzymatic activities of alanine amino transaminase (ALT) and aspartate amino transaminase (AST) were detected using assay kits supplied by Jiancheng Bioengineering Institute (A154-1-1, C011-2-1, C009-2-1 and C010-2-1, Nanjing, China).

### Statistics

The data in this study are expressed as the mean ± SD. All experimental procedures, treatment, and data analyses were performed in a blinded manner. The quantitative analysis of immunoblots and mRNA expression was normalized to reduce baseline variability between independent experiments. Two-group comparisons were completed using a 2-tailed Student’s t-test; 3 or more different group comparisons were analyzed by one-way ANOVA. A comparison was considered significant if a *p* value was less than 0.05. All statistical analyses were performed using GraphPad Prism 9.

## Results

### Effect of Myr on the viability of RA FLSs

**FIGURE 1:**
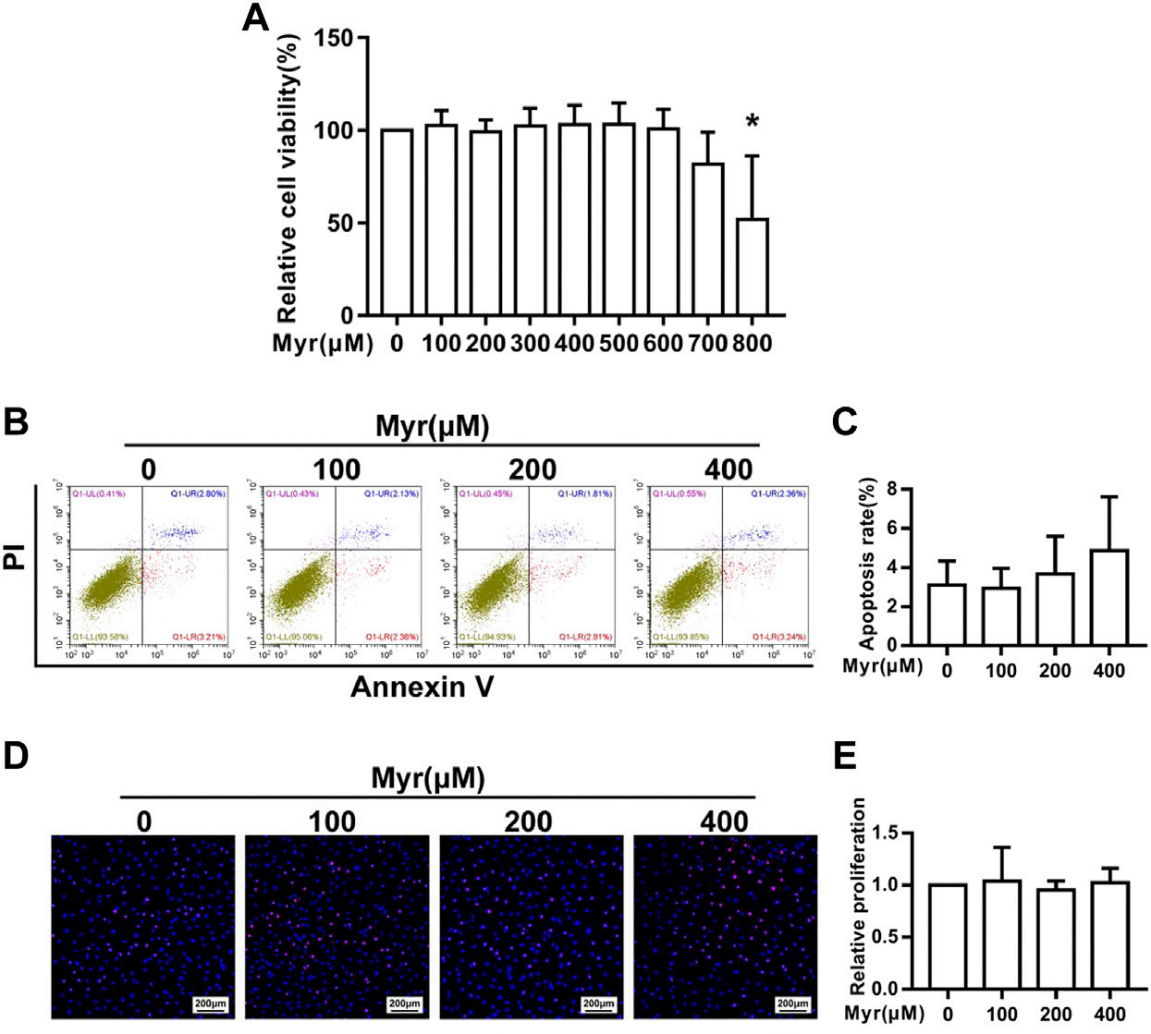
Effect of Myr on the viability, proliferation and apoptosis of RA FLSs. The cells were treated with Myr at different concentrations (0–800 μM) for 24 h. **(A)**The CCK-8 assay was used to detect the cell viability. **(B,C)** Effect of Myr on the apoptosis of RA FLSs. Cell apoptosis was detected by a flow cytometry-based Annexin V-APC/PI assay. Representative images are shown. **(D,E)** Effect of Myr on the proliferation of RA FLSs. Cell proliferation was assessed by the EdU assay. Representative images are shown (original magnification, ×100). The data are expressed as the mean ± SD from at least 3 independent experiments. ^*^ *p* < 0.05 versus DMSO group.

To evaluate the viability of RA FLSs treated with Myr, we assessed RA FLS viability by the CCK-8 assay. RA FLSs were treated with various concentrations of Myr (0, 100, 200, 300, 400, 500, 600, 700, and 800 μM) for 24 h. The results showed that 700 μM Myr did not significantly influence RA FLS viability (Figure 1A). To exclude the cytotoxic effects of Myr, we selected concentrations of 100, 200, and 400 μM to further evaluate the effect of Myr on the abnormal behaviors of RA FLSs.

### Effect of Myr on the apoptosis and proliferation of RA FLSs

Abnormal apoptosis resistance and hyperplasia of RA FLSs contribute to synovial hyperplasia. Thus, we explored the role of Myr in apoptosis and proliferation of RA FLSs. We first evaluated the apoptosis of RA FLSs by staining Annexin V-APC/PI and detecting by flow cytometry. Flow cytometry detection found that Myr treatment at concentrations of 100, 200, and 400 μM for 24 h did not increase RA FLS apoptosis (Figures 1B,C). Then, we used an EdU assay to determine the role of Myr treatment on the RA FLSs proliferation. The result showed that the proliferation of RA FLSs was not influenced by treatment with Myr at concentrations of 100, 200, and 400 μM (Figures 1D,E). These data suggest that Myr does not influence the proliferation and apoptosis of RA FLSs.

### Myr inhibited the migration and invasion of RA FLSs

**FIGURE 2:**
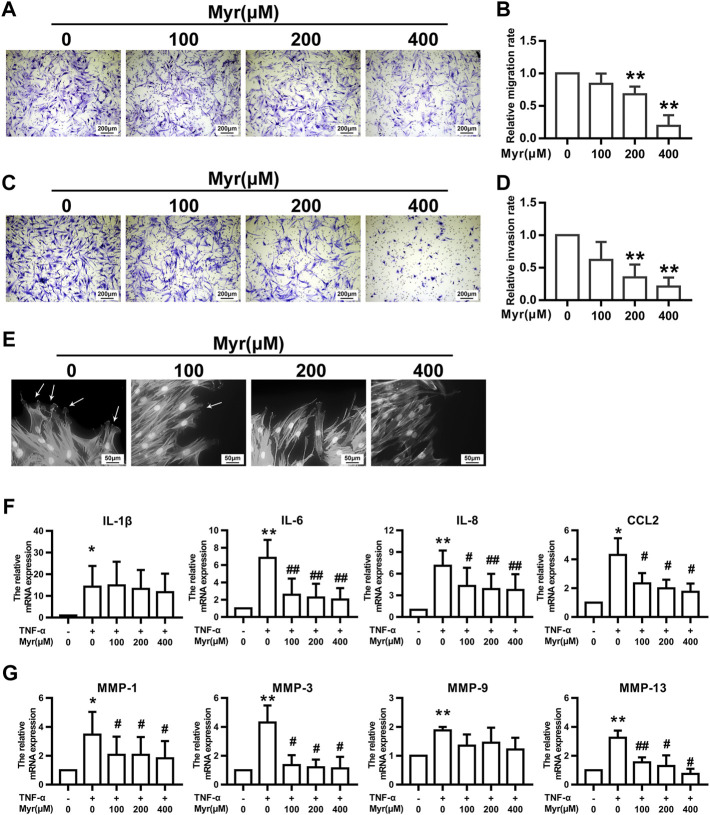
Myr inhibits the migration, invasion, and TNF-α–induced expression of proinflammatory cytokines and MMPs of RA FLSs. The cells were pretreated with DMSO or Myr (100, 200, and 400 μM) for 24 h **(A–D)** The migration and invasion of RA FLSs were evaluated by a Boyden chamber assay. Transwell inserts coated with a Matrigel basement membrane matrix were used to detect the invasion of RA FLSs. The relative migration or invasion rate was calculated by counting migrated or invaded cells and then normalized to that in DMSO group. Representative images (original magnification, ×100) are shown. Graphs show the relative migration **(B)** and invasion **(D)** rates. **(E)** Myr impaired lamellipodia formation of RA FLSs. Arrows indicate lamellipodia formation. Representative images are shown (original magnification, ×400). **(F,G)** The cells were preincubated with DMSO, DMSO with TNF-α (10 ng/ml) or Myr (100, 200, and 400 μM) with TNF-α for 24 h. RT-qPCR was used to measure the expression of proinflammatory cytokines **(F)** and MMPs **(G)** in RA FLSs. Data **(B,D,F,G)** show the mean ± SD of samples from at least 3 independent experiments. ^*^ *p* < 0.05, ^**^ *p* < 0.01 versus DMSO; ^#^ *p* < 0.05, ^##^ *p* < 0.01 versus TNF-α+DMSO.

RA FLS migration and invasion properties play an essential role in disease progression, which can occur continuously in noninflammatory environments. Thus, we chose a transwell migration assay to determine the potential function of Myr on the migration ability of RA FLSs. Cells were pretreated with Myr at multiple concentrations (0, 100, 200, and 400 μM) for 24 h. The results showed that Myr at concentrations of 200 and 400 μM inhibited RA FLSs migration in a dose-dependent manner (Figures 2A,B).

Reminiscent of cancer cells, RA FLSs exhibit invasive characteristics that are associated with cartilage and bone erosion during RA (You et al., 2014). Thus, we used Matrigel-coated Transwell membranes to evaluate RA FLS invasion. In the presence of Myr, RA FLS invasion tended to decrease in a dose-dependent manner (Figures 2C,D).

Cytoskeletal reorganization is critical for cell morphology and mobility, and we investigated the impact of Myr on actin in migrating cells after wounding. Fluorescence phalloidin staining showed that Myr suppressed the reorganization of F-actin and the formation of lamellipodia (Figure 2E).

### Myr reduced proinflammatory cytokine and MMP expression in RA FLSs induced by TNF-*α*

Since proinflammatory cytokines and MMPs contribute to synovial inflammation and joint damage in RA patients, we examined the impact of Myr on the expression of IL-1*β*, IL-6, IL-8, CCL2, MMP-1, MMP-3, MMP-9, and MMP-13 in RA FLSs through RT-qPCR. In response to TNF-*α*, the mRNA levels of the above cytokines and MMPs in RA FLSs increased compared to those in the control group. Treatment with Myr inhibited the increased expression of IL-6, IL-8, CCL2, MMP-1, MMP-3, and MMP-13 induced by TNF-α in a dose-dependent manner (Figures 2F,G).

### AIM2 is required for the inhibitory role of myr in RA FLS function

**FIGURE 3:**
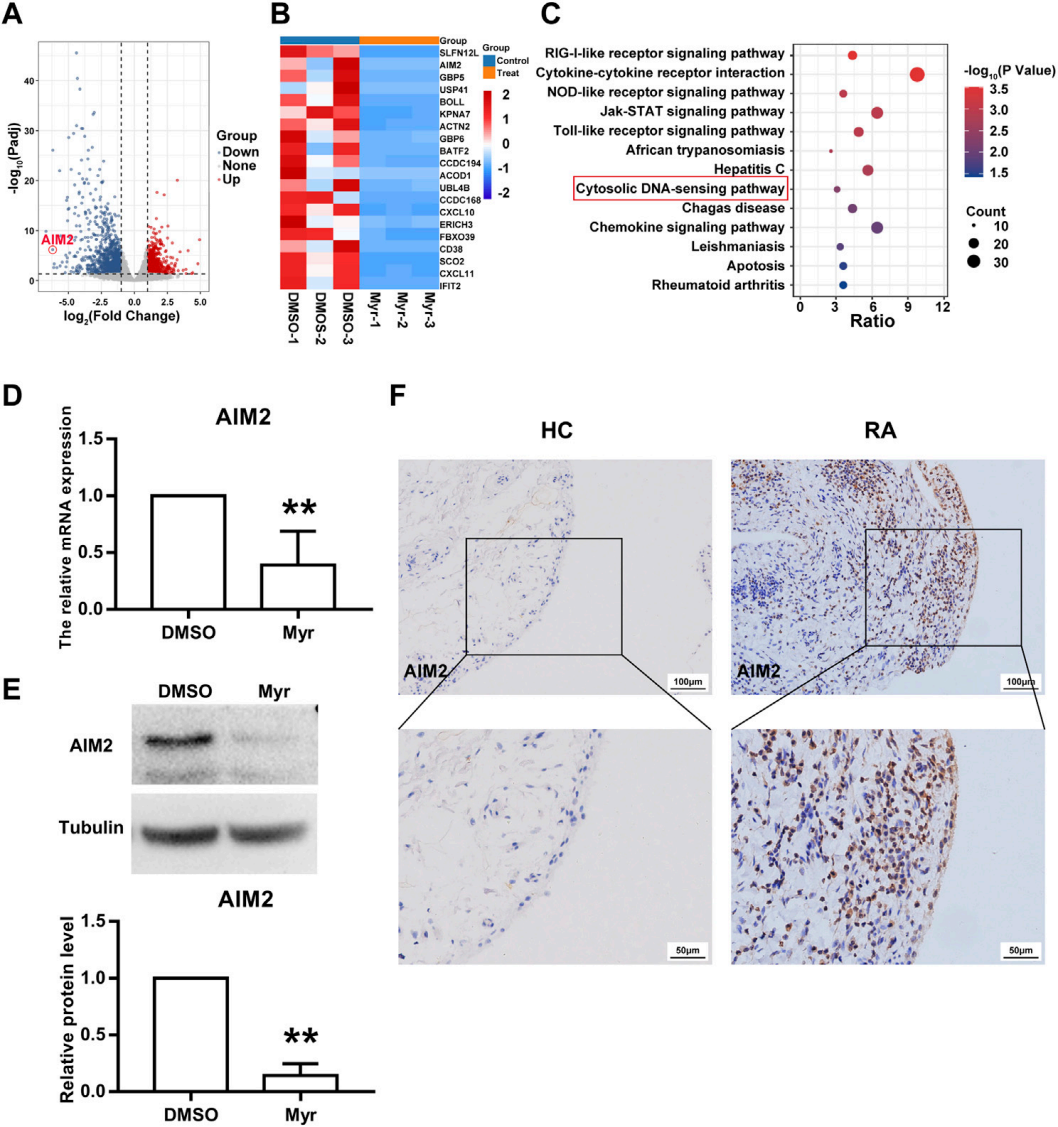
AIM2 is identified as a target of Myr. **(A)** Volcano plot indicated up-regulated and down-regulated gene (Padj<0.05 and |log_2_FC| >1) by RNA sequencing in Myr-treated versus DMSO-treated RA FLSs from 3 patients. **(B)** Heatmap of the top 20 differentially expressed genes (DEGs). **(C)** Bubble plot of the top 13 enriched KEGG pathways of DEGs. **(D,E)** Myr downregulated the gene and protein expression of AIM2 by using RT-qPCR **(D)** and Western blot **(E)**. Data are presented as the mean ± SD from at least 3 independent experiments. **(F)** Immunohistochemistry staining was used to measured AIM2 expression in synovial tissues from RA patients and HC subjects. Original magnification, ×200. ^**^ *p* < 0.01 versus HC.

We further explored the mechanism by which Myr regulates the biological function of RA FLSs. RNA sequencing was used to identify the differentially expressed transcriptome of Myr-treated RA FLSs compared with the DMSO control. An adjusted *p*-value (Padj) < 0.05 was used to determine the differential expressed gene. By analyzing the RNA-seq data, we identified a total of 769 upregulated genes and 1,187 downregulated genes (with Padj<0.05 and |log_2_FC| >1) in the 400 μM Myr treatment group compared with the DMSO control (Figure 3A). A detailed list of the differentially expressed genes is shown in Supplementary Data Supplementary Table S3. Figure 3B shows the top 20 downregulated genes. For the top 5 downregulated genes, there was discordance between RNA-seq and RT-qPCR data in the relative expression levels of SLFN12L. Thus, we focused on AMI2, the second-ranked gene among the downregulated genes, with Myr treatment. In addition, KEGG analysis demonstrated that the downregulated genes were enriched in pathways such as the RIG-like receptor signaling pathway, cytokine–cytokine receptor interaction, NOD-like receptor signaling pathway, JAK-STAT signaling pathway, and cytosolic DNA-sensing pathway (Figure 3C). AIM2, as a cytosolic DNA sensor, has important roles in many biological functions (Wang B. et al., 2020; Zhu et al., 2021).

We used RT-qPCR and Western blotting to verify the reliability of RNA-seq. The results showed a significant decrease in AIM2 gene and protein expression in Myr-treated RA FLSs compared to the DMSO control (Figures 3D,E). To further explore the function of AIM2 in abnormal RA FLS behavior, we first compared the expression of AIM2 in synovial tissues and FLSs from RA patients with those from HC controls. As Figure 3F shows, AIM2 expression increased in RA synovium. Moreover, as shown in Supplementary Figure S1, the mRNA and protein levels of AIM2 in RA FLSs *in vitro* also increased, which indicated that AIM2 may be an important regulator of the inhibitory biological functions of RA FLSs.

Next, we used siRNA oligonucleotide sequences to downregulate the expression of AIM2 and further investigate the role of AIM2 in regulating RA FLS function. Both RT-qPCR and WB results showed that siRNA-1 and siRNA-3 against AIM2 obviously decreased the expression of AIM2 (Supplementary Figure S2). Thus, siRNA-1 and siRNA-3 were chosen for subsequent experiments.

**FIGURE 4:**
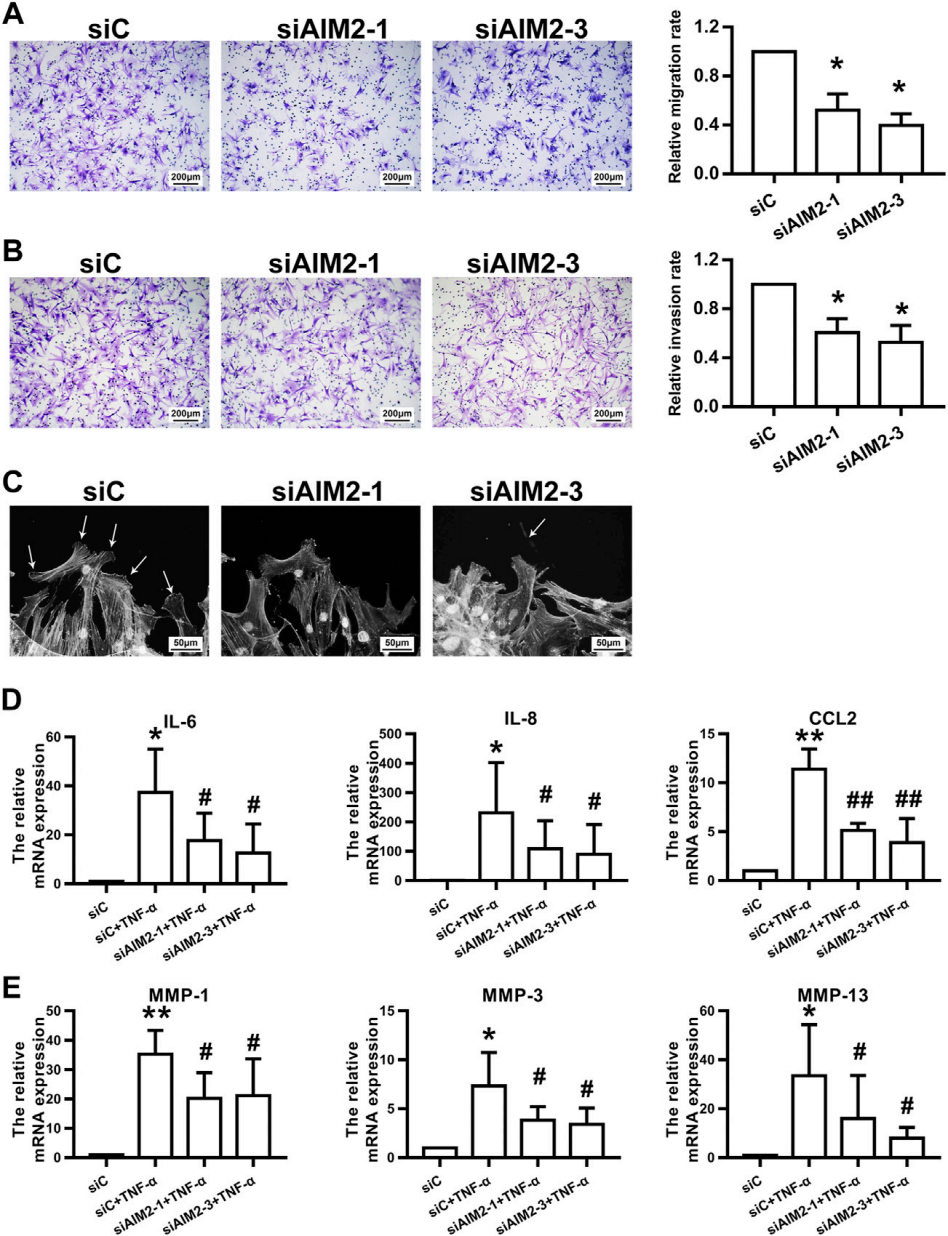
AIM2 knockdown regulated RA FLS migration and invasion. RA FLSs were transfected with siRNAs against AIM2 (siAIM2-1, siAIM2-3) or control siRNA (siC). **(A,B)** Effect of AIM2 knockdown on the migration and invasion of RA FLSs. RA FLS migration **(A)** and invasion **(B)** were measured with a Boyden chamber assay. The relative migration and invasion rates were calculated by counting migrated or invaded cells and then normalized to that in control group. Representative images (original magnification, ×100) are shown. Data show the mean ± SD of samples from at least 3 independent experiments. **(C)** Effect of AIM2 knockdown on lamellipodia formation of RA FLSs. White arrows indicate lamellipodia formation. **(D,E)** AIM2 knockdown downregulated TNF-α–induced expression of proinflammatory cytokines and MMPs. RT-qPCR was used to detect the expression of cytokines **(D)** and MMPs **(E)**. Data show the mean ± SD from at least 3 independent experiments. ^*^ *p* < 0.05, ^**^ *p* < 0.01 versus siC; ^#^ *p* < 0.05, ^##^ *p* < 0.01 versus TNF-α + siC.

As shown in Figures 4A–C, the migration (A) and invasion (B) of RA FLSs were inhibited upon AIM2 knockdown. AIM2 knockdown also impaired F-actin reorganization and lamellipodium formation (C). In addition, we found that AIM2 knockdown also reduced the expression of IL-6, IL-8, CCL2, MMP-1, MMP-3, and MMP-13 induced by TNF-α in RA FLSs (Figures 4D,E). These data suggested that AIM2 participated in regulating the biological function of RA FLSs.

### AIM2 regulates RA FLS functions *via* AKT phosphorylation

**FIGURE 5:**
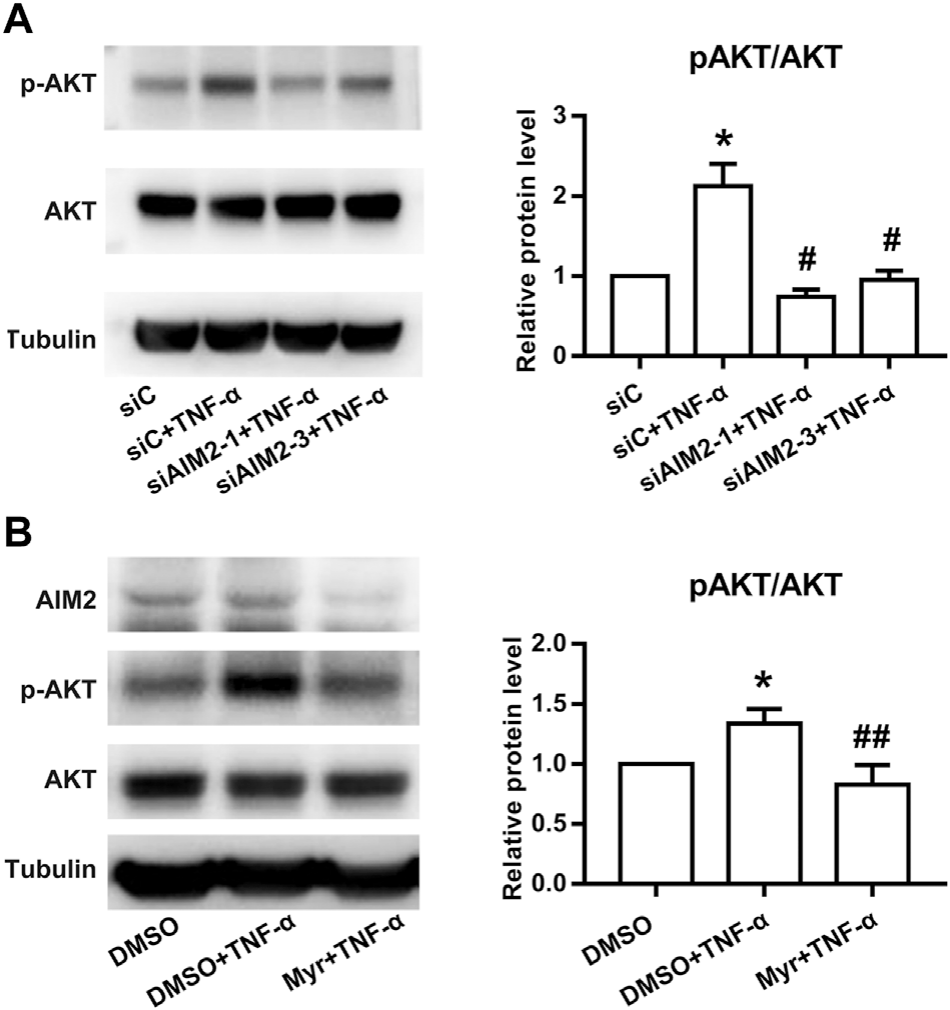
Effect of Myr treatment or AIM2 knockdown on the activation of the AKT pathway in RA FLSs. Protein expression was detected using Western blot analysis. **(A)** RA FLSs were transfected with siRNAs for AIM2 (siAMI2-1, siAIM2-3) or control siRNA (siC) for 72 h and then stimulated with TNF-α (10 ng/ml) for 30 min **(B)** RA FLSs were treated with Myr (400 μM) or DMSO for 24 h, and then stimulated with TNF-α (10 ng/ml) for 30 min. Data **(A,B)** show the mean ± SD from at least 3 independent experiments. ^*^ *p* < 0.05 versus siC or DMSO, ^#^ *p* < 0.05, ^##^ *p* < 0.01 versus siC + TNF-α or DMSO + TNF-α.

Previous studies have shown that Myr exerts anti-inflammatory effects by inhibiting the activation of AKT (Schwanke et al., 2013). In addition, AIM2 was also reported to regulate the activation of PI3K/AKT in other cell lines (Zheng et al., 2022). Thus, we investigated whether AIM2 modulates RA FLS functions by regulating AKT activation. Our results showed that AIM2 knockdown decreased AKT phosphorylation induced by TNF-α (Figure 5A). In addition, we also observed that Myr blocked the phosphorylation of AKT induced by TNF-α (Figure 5B). These results suggest that Myr regulates RA FLS functions by suppressing the AIM2/AKT axis.

### Myr has a therapeutic effect on collagen-induced arthritis

**FIGURE 6:**
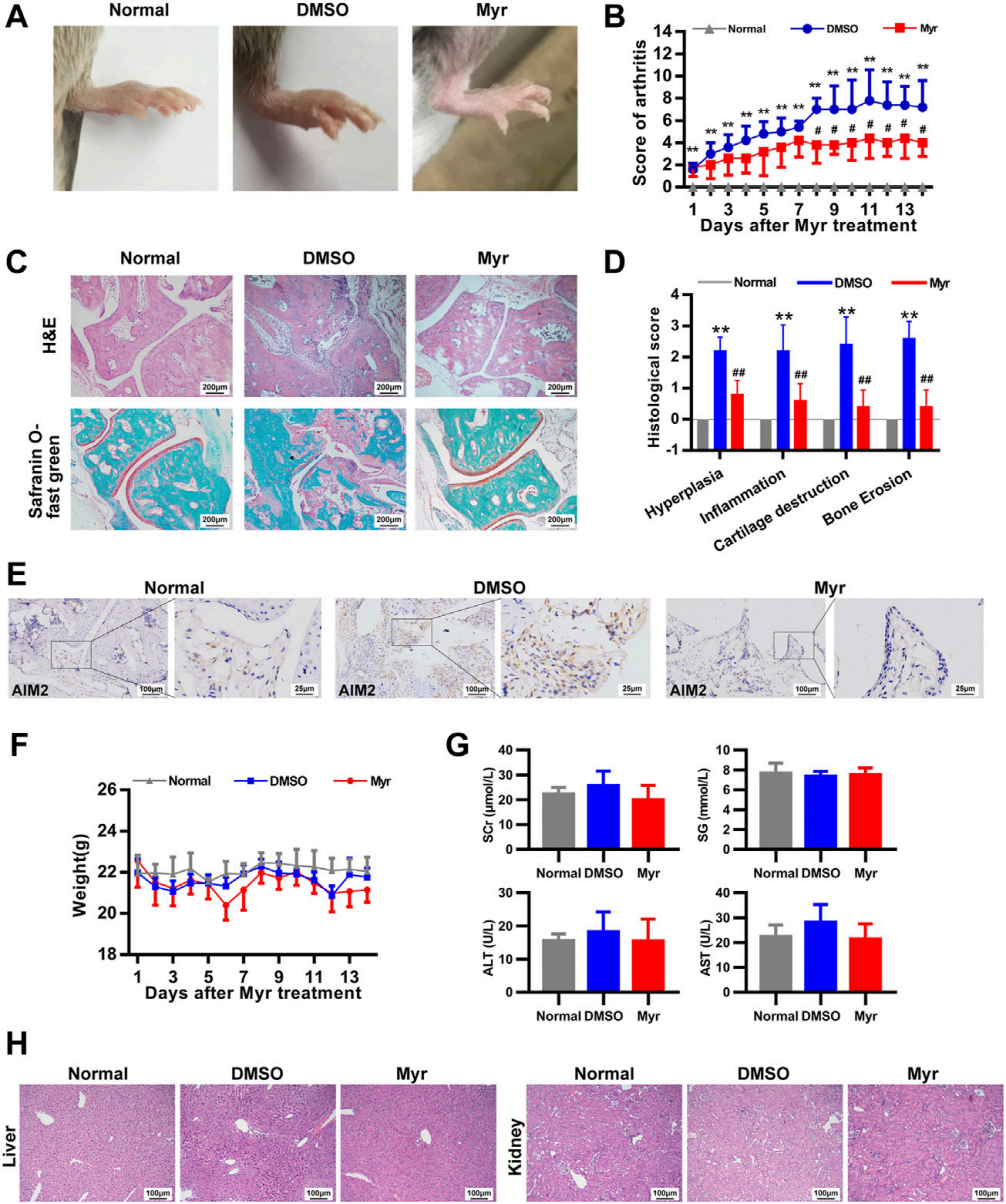
Myr reduces the severity of arthritis in mice with CIA. CIA mice were gavaged with Myr (100 mg/kg/d, *n* = 5) or 1%DMSO (as a model control, n = 5) daily for 14 days, normal mice were presented as normal control (normal). **(A,B)** Myr attenuated clinical symptom in CIA mice. The values in **(B)** are the mean ± SD of clinical scores in normal mice or mice treated with Myr or DMSO. **(C,D)** H&E and Safranin O-Fast green staining was used to evaluate synovial hyperplasia, inflammation, cartilage destruction, and bone erosion. Original magnification, ×100. The graph **(D)** indicates the mean ± SD scores for synovial hyperplasia, inflammation, cartilage destruction, and bone erosion. **(E)** Immunohistochemical staining was used to measure the expression of AIM2 in synovial tissues from mice. Representative images were from normal mice, DMSO-treated mice, and Myr-treated mice (original magnification, × 200). **(F)** Effect of Myr on Body weight in mice. Data were shown as the mean ± SD. **(G)** Effect of Myr on the levels of serum glucose (SG), serum creatinine (SCr), and alanine aminotransferase (ALT) and aspartate aminotransferase (AST) in mice. Data were shown as the mean ± SD. **(H)** Representative images show the pathological changes of the liver and kidney in mice. Graph indicates the mean ± SD from 5 mice. Original magnification, ×200. ^*^ *p* < 0.05, ^**^ *p* < 0.01 versus Normal, ^#^ *p* < 0.05, ^##^ *p* < 0.01 versus DMSO.

To explore the effect of Myr *in vivo*, we used CIA as the animal model of RA. Mice were intragastrically administered Myr or 1% DMSO for 14 days. As shown in Figures 6A,B, Myr treatment reduced clinical scores compared with the DMSO control. H&E staining and safranin O-fast green staining showed decreased synovial hyperplasia, inflammatory cell infiltration, and cartilage and bone destruction of the joints in Myr-treated mice (Figures 6C,D). Immunohistochemical results showed that AIM2 expression in the synovium was downregulated in Myr-treated mice (Figure 6E). In addition, we further detected the expression of inflammatory cytokines and MMPs in synovium from mice with CIA. The results showed that Myr treatment reduced the IL-6, IL-8, CCL2, MMP-1, MMP-3 and MMP-13 expression in synovium (Supplementary Figure S3). Meantime, we also found that the p-AKT level in the synovium was decreased in Myr treatment group compared to DMSO control group (Supplementary Figure S4).

We further clarified the toxic effect of Myr *in vivo*. There was no significant difference in body weight between the Myr-treated and DMSO-treated mice during the experiment (Figure 6F). We further tested the biochemical indicators in the peripheral blood of mice. There were no significant changes in liver parameters, including alanine aminotransferase (ALT) and aspartate aminotransferase (AST) levels, renal parameters (serum creatinine levels), or serum glucose levels in mice treated with Myr (Figure 6G). In addition, we further detected histopathological alterations in the kidneys and livers, which were not significantly different in either group (Figure 6H). These results indicate the safety of Myr treatment in CIA mice.

## Discussion

Our results showed Myr regulates the inflammatory response, migration, and invasion of RA FLSs. We further found that AIM2 mediates the role of Myr in regulating RA FLS functions. Additionally, Myr treatment ameliorated the symptoms of arthritis in CIA mice. Therefore, these data indicate that Myr ameliorated synovial inflammation and bone erosion by regulating AIM2 function in RA FLSs and might have a potential therapeutic effect on RA.

Although the current treatment of RA has improved, there are still many patients with poor responses to treatment (Ai et al., 2018). RA FLSs display an aggressive phenotype by promoting inflammation, invading cartilage and destroying bone (Bottini and Firestein, 2013). Growing evidence suggests that direct targeting of FLS may be a viable therapeutic strategy for the treatment of RA (Ai et al., 2018; Shen et al., 2021). In this study, we found that Myr inhibits the migration and invasion of RA FLSs *in vitro*. Furthermore, our results also demonstrated that Myr treatment downregulated the expression of MMP-1, MMP-3, and MMP-13, which corroborated the regulatory effect of Myr on RA FLS invasion. Myr has been reported to significantly inhibit cell migration and reduce the matrix metalloproteinase (MMP) activity of vascular endothelial cells (Hu et al., 2020). In addition, it has been shown that Myr inhibits the inflammatory response by attenuating the phosphorylation of the AKT signaling pathway in experimental colitis and osteoclastogenesis (Schwanke et al., 2013; Wang et al., 2018). Our results also showed that Myr treatment downregulated the expression of CCL2, IL-6, and IL-8 in response to TNF-ɑ treatment. Moreover, Myr administration showed an inhibitory effect on synovial inflammation and articular bone and cartilage destruction in CIA mice. In conclusion, Myr has a potential therapeutic effect for alleviating inflammation and destruction of synovial joints in RA.

We used RNA sequencing analysis to investigate the underlying mechanisms of how Myr regulates RA FLS functions. Activation of absent in melanoma 2 (AIM2) was identified, which might be a new target of Myr in RA FLSs. AIM2 is involved in the inflammasome-forming process in response to cytosolic dsDNA (Cinat et al., 2021). In a previous study, AIM2 inflammasome activation regulated many cellular processes, including cell pyroptosome formation (Pastar et al., 2021), cell differentiation (Yang et al., 2021), and migration (Dawson et al., 2021). For example, the AIM2 inflammasome was activated in primary human keratinocytes due to intracellular *S. aureus*, which promoted cell pyroptosis and deregulation of the inflammatory response in nonhealing diabetic foot ulcers (Pastar et al., 2021). In another study, it was shown that AIM2 overexpression augmented the tumor load of human GC cell line xenografts by interacting with EB1 to promote epithelial cell migration and tumorigenesis, which was independent of inflammasome activity and inflammation (Dawson et al., 2021). More importantly, it was reported that AIM2 was increased in RA synovium compared to osteoarthritis (OA) patients. In addition, AIM2 knockdown suppresses the proliferation of RA FLSs but not cell migration and apoptosis (Chen et al., 2020). Herein, we observed some different results. Compared to HC group, increased expression of AIM2 was also observed in synovial tissues from patients with RA. We also found that AIM2 expression was increased in RA FLSs. Although, the age of HC group mismatch with that of RA group, the AIM2 mRNA and protein expression showed that there is no significant difference in different age groups. Our results showed that Myr treatment and AIM2 knockdown reduced cell migration, invasion, and expression of MMP-1, MMP-3, and MMP-13. Interestingly, AIM2 knockdown also downregulated the TNF-α-stimulated expression of CCL2, IL-6, and IL-8 in RA FLSs, which is consistent with the effect of Myr on RA FLSs. Moreover, Myr treatment reduced synovial AIM2 expression in CIA mice. Therefore, our results demonstrate that AIM2 mediates the inhibitory function of Myr in regulating RA FLS behaviors. However, because Myr is a small molecule natural agent that has broad pharmacological effects, we do not exclude that other unknown factor may have affected abnormal RA-FLS behaviors.

It was reported that oral administration of Myr inhibited dextran sulfate sodium (DSS)-induced experimental colitis in mice by regulating the activation of AKT (Schwanke et al., 2013). In addition, AIM2 also showed an inhibitory effect on the proliferation, invasion, and migration of osteosarcoma cells by reducing AKT phosphorylation (Zheng et al., 2022). Thus, we further explored whether AIM2 mediates the Myr-induced inhibitory effect on abnormal RA FLS functions by regulating the PI3K/AKT signaling pathway. Our results demonstrated that Myr decreased the phosphorylation of AKT induced by TNF-α. AIM2 knockdown also mimic inhibitory effect of Myr on AKT activation. Indeed, the regulatory function of the AKT signaling pathway in synovial inflammation and aggression in RA has been reported previously (Bartok et al., 2012; Malemud, 2013; Sugiura et al., 2020). These data suggest that AIM2 regulates Myr-induced repression of RA FLS functions by modulating AKT activation.

In conclusion, our study show that Myr treatment ameliorated inflammatory responses and the aggressive phenotype of RA FLSs, reducing AIM2 expression, sequentially inhibiting AKT phosphorylation. These suggest that Myr has an inhibitory effect on rheumatoid synovial inflammation and joint destruction caused by FLS, and serves as a potential therapeutic drug for RA.

## Data Availability

The RNA-seq data for this study can be found in (https://www.ncbi.nlm.nih.gov/geo/query/acc.cgi?&acc=GSE198593).
