# Supplementary material for: Myricitrin inhibits fibroblast-like synoviocyte-mediated rheumatoid synovial inflammation and joint destruction by targeting AIM2
Source: Front Pharmacol. 2022 Aug 31;13:905376. doi: 10.3389/fphar.2022.905376 (PMC9471193; doi:10.3389/fphar.2022.905376)
Supplement: Supplementary file 2 [file DataSheet1.pdf]

Supplementary data

Supplementary Fig. S1: Increased levels of AIM2 in RA FLSs. The expression of AIM2 in RA FLSs and HC FLSs was measured by RT-qPCR (**A**) and western blot (**B**). Data show the mean ± SD from at least 3 independent experiments. *P < 0.05 versus HC.

Supplementary Fig. S2: The efficiency of AIM2 knockdown. RA FLSs were subjected to RT-qPCR analysis (A) and Western blotting (B). A representative blot is shown. The data show the mean ± SD of samples from at least 3 independent experiments. *P < 0.05, **P < 0.01 vs. CTR.

Supplementary Fig. S3: Immunohistochemical staining was used to measure the expression of IL-6, IL-8, CCL2, MMP-1, MMP-3, MMP-13 in synovial tissues from CIA mice. Representative images were from normal mice, DMSO-treated mice, and Myr-treated mice (original magnification, ×200).

Supplementary Fig. S4: Immunohistochemical staining was used to measure the expression of p-AKT in synovial tissues from mice. Representative images were from normal mice, DMSO-treated mice, and Myr-treated mice (original magnification, ×200).

Supplementary Fig. S5. Detection of surface molecules and phagocytosis of FLSs. The surface molecules CD11b, FcγRII, and FcγRIII receptor were detected by flow cytometry(A). Phagocytosis of FLSs were evaluated by fluorescent bead uptake assay. Representative images were shown(B) (original magnification, ×100).

Fig. S1

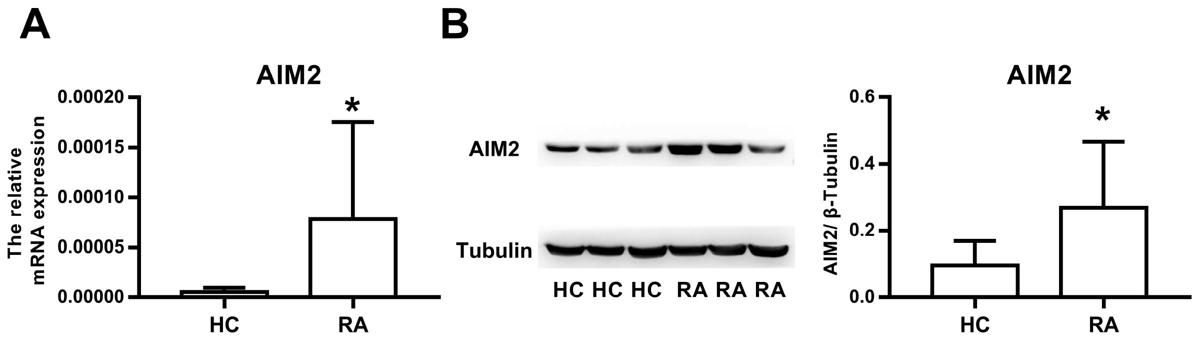
A
AIM2
The relative mRNA expression
0.00020
0.00015
0.00010
0.00005
0.00000
*
HC
RA
B
AIM2
Tubulin
HC HC HC RA RA RA
AIM2
AIM2/ β-Tubulin
0.6
0.4
0.2
0.0
*
HC
RA

Fig. S2

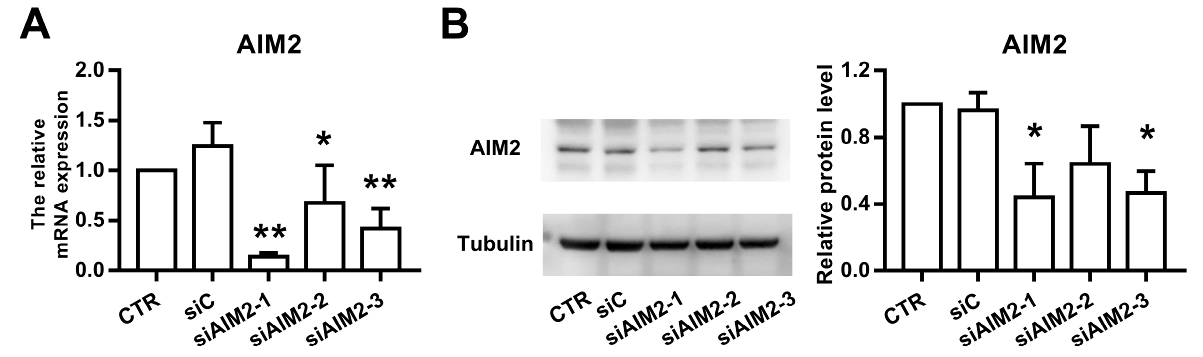
A
AIM2
The relative mRNA expression
2.0
1.5
1.0
0.5
0.0
**
*
**
CTR
siC
siAIM2-1
siAIM2-2
siAIM2-3
B
AIM2
Tubulin
CTR
siC
siAIM2-1
siAIM2-2
siAIM2-3
AIM2
Relative protein level
1.2
0.8
0.4
0.0
*
*
CTR
siC
siAIM2-1
siAIM2-2
siAIM2-3

Fig. S3

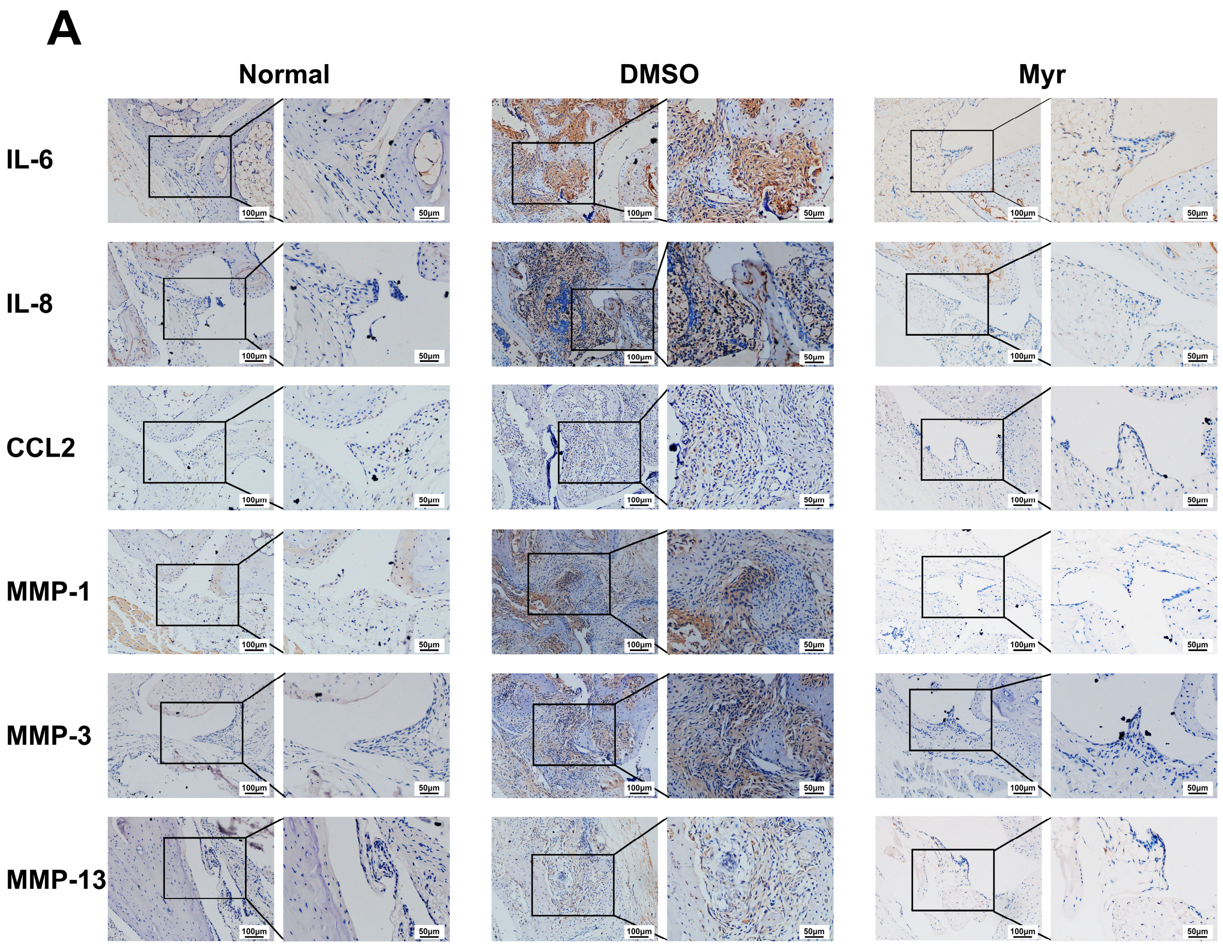

Fig. S4

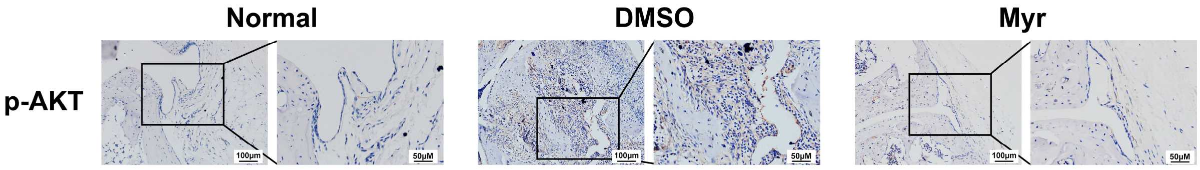

Normal
DMSO
Myr
p-AKT
100μm
50μM

Fig. S5

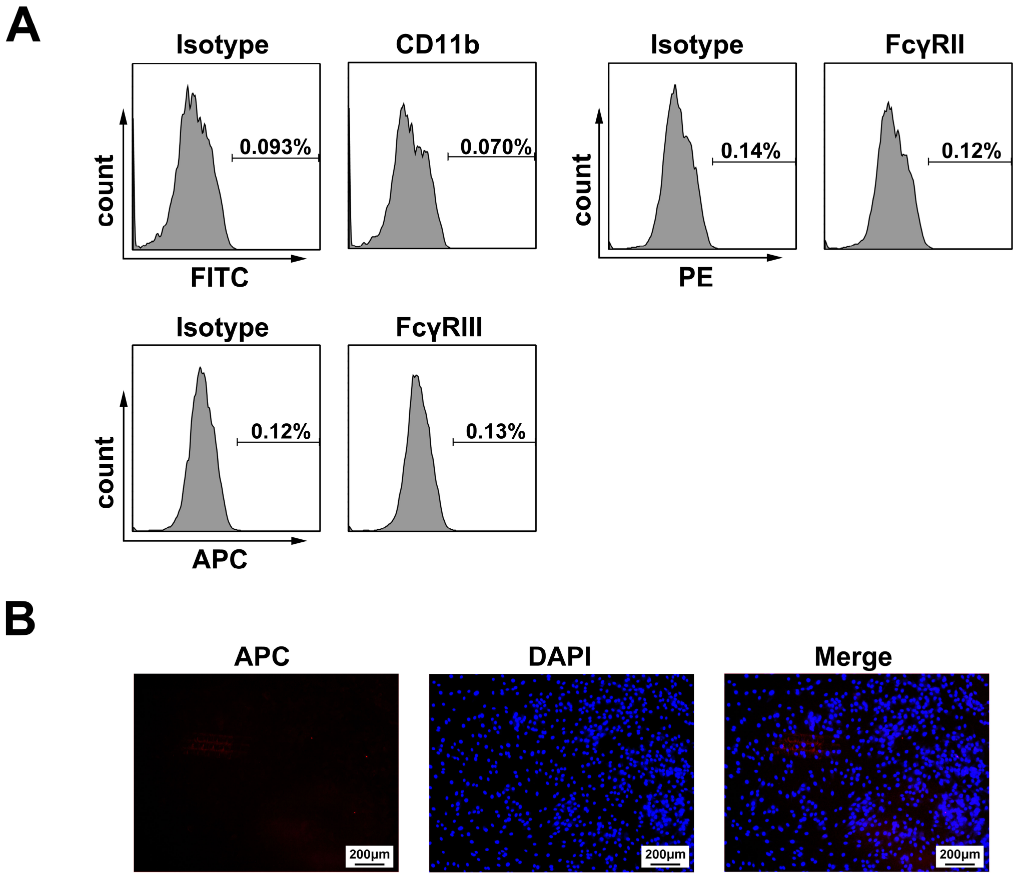

A
Isotype
CD11b
Isotype
FcγRII
0.093%
0.070%
0.14%
0.12%
count
FITC
PE
Isotype
FcγRIII
0.12%
0.13%
APC
B
APC
DAPI
Merge
200μm

Table S1: Sequences of primers for RT-qPCR.

gene		sequences
AIM2	Forward	AGCAAGATATTATCGGCACAGTG
	Reverse	GTTCAGCGGGACATTAACCTT
β-actin	Forward	TCAAGATCATTGCTCCTCCTGAG
	Reverse	ACATCTGCTGGAAGGTGGACA
IL-1β	Forward	ATGATGGCTTATTACAGTGGCAA
	Reverse	GTCGGAGATTCGTAGCTGGA
IL-6	Forward	ACTCACCTCTTCAGAACGAATTG
	Reverse	CCATCTTTGGAAGGTTCAGGTTG
IL-8	Forward	ACTGAGAGTGATTGAGAGTGGAC
	Reverse	AACCCTCTGCACCCAGTTTTC
CCL2	Forward	CAGCCAGATGCAATCAATGCC
	Reverse	TGGAATCCTGAACCCACTTCT
MMP-1	Forward	CTCTGGAGTAATGTCACACCTCT
	Reverse	TGTTGGTCCACCTTTCATCTTC
MMP-3	Forward	AAAGACAGGCACTTTTGGCG
	Reverse	CTTCATATGCGGCATCCACG
MMP-9	Forward	TGTACCGCTATGGTTACACTCG
	Reverse	GGCAGGGACAGTTGCTTCT
MMP-13	Forward	ACTGAGAGGCTCCGAGAAATG
	Reverse	GAACCCCGCATCTTGGCTT

Table S2: The sequences of siRNA oligonucleotides.

AIM2	siRNA-1	GCAACGTGCTGCACCAAAA
	siRNA-2	GCAGGAATCTATCAGAGAA
	siRNA-3	GAAGCGCTGTTTGCCAGTT

Table S4. Demographic and clinical features of patients with active RA

Age, yrs (mean ± SD)	56.67±4.63
Female: Male	8:2
Disease duration, yrs (mean±SD)	11.90±6.92
Rheumatoid factor-positive (%)	90
Anti-CCP-positive (%)	90
DAS28 (mean±SD)	6.09±1.20
Previous medications (%)	
Prednisone (<10mg/d)	30
Methotrexate	70
Leflunomide	10
Sulfasalazine	0
Hydroxychloroquine	20
NSAIDs	50

Table S5. Demographic and clinical features of health control subjects

Age, yrs (mean ± SD)	37.55±13.33
Female: Male	3:7
Previous medications (%)	
Prednisone (<10mg/d)	0
Methotrexate	0
Leflunomide	0
Sulfasalazine	0
Hydroxychloroquine	0
NSAIDs	80
